# Dwarfs in disguise: multiple spinal abscesses and spondylodiscitis caused by an *Enterococcus faecium* small-colony variant

**DOI:** 10.1099/acmi.0.000012

**Published:** 2019-03-27

**Authors:** Steffen Höring, Katharina Sobotta, Sylke Schneider, Bettina Löffler, Jürgen Rödel

**Affiliations:** 1 Institute of Medical Microbiology, Jena University Hospital, Am Klinikum1, D-07747 Jena, Germany; 2 Clinic for Internal Medicine, Waldkrankenhaus ‘Rudolf Elle’, Klosterlausnitzer Straße 81, D-07607 Eisenberg, Germany

**Keywords:** small-colony variant, SCV, *Enterococcus faecium*, spondylodiscitis, spinal abscess

## Abstract

Small-colony variants are slow-growing subpopulations of bacteria known to be involved in latent or recurrent infections, especially in deep-seated foci. Their atypical growth in small colonies can hamper prompt and correct identification in clinical specimens. Here, we present the first case of multiple spinal abscesses and spondylodiscitis associated with an *Enterococcus faecium* small-colony-variant in an immunocompetent patient. This case demonstrates the diagnostic challenges when encountering this phenotype in the diagnostic laboratory.

## Introduction

Small-colony variants (SCVs) are slow-growing subpopulations of bacteria known to be involved in latent or recurrent infections. By lowering their virulence and metabolic activity, SCVs escape the host immune defence and are able to persist in deep-seated foci [1]. Their atypical growth in small colonies on agar medium can hamper prompt and correct identification when cultivated from clinical specimen. While SCV infections are well described for various bacteria, such as S*taphylococcus aureus* or *Pseudomonas aeruginosa ,* only few clinical case reports exist so far for enterococci [1–7]. Here, we present the first case of multiple spinal abscesses and spondylodiscitis associated with an *Enterococcus faecium* SCV in an immunocompetent patient. This case underlines the importance of SCVs in deep-seated infections and demonstrates the diagnostic challenges experienced when encountering this phenotype in the diagnostic laboratory.

## Clinical case

A 75-year-old male patient presented at our emergency department with acute febrile illness and worsening of general condition. Physical examination yielded febrile body temperature (39.1 °C) and pain on percussion over the lower back. The underlying diseases were a chronic obstructive pulmonary disease due to chronic nicotine abuse (50 pack years), mild dementia and a history of alcohol abuse considered as a predisposing factor for infectious diseases. Furthermore, a compression fracture of the 12th thoracic vertebral body associated with osteoporosis was described in the previous medical history. Basic laboratory testing revealed a mild normocytic anaemia, mild leukocytosis (11.200 cells/l), thrombocytosis (406.000 cells/l) and elevated serum levels of C-reactive protein (143.6 mg l^−1^). Urinary tract infection was ruled out by urine dipstick test and chest radiography showed no pulmonary focus of infection. Blood cultures were drawn before the initiation of intravenous antibiotic therapy with amoxicillin/clavulanate 875 mg/125 mg BID.

**Fig. 1.**
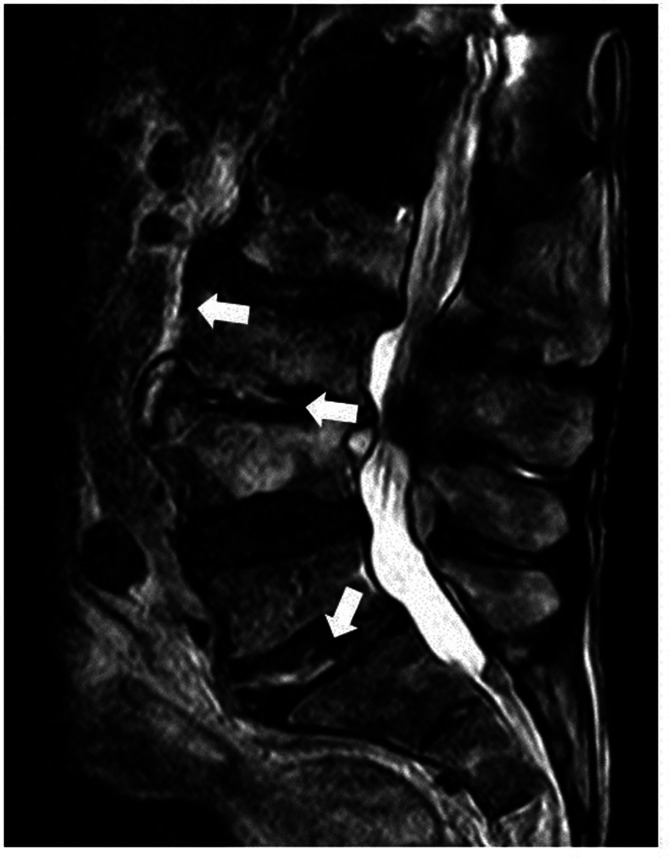
T2-weighted sagittal magnetic resonance imaging (MRI) scan of the lumbosacral spine. Hyperintense signals within the L3/L4 and the L5/S1 disc space and praevertebral L3 represent fluid accumulations.

On day two of treatment, four out of four blood culture flasks were flagged positive and Gram-staining showed Gram-positive cocci. Final blood culture results, including pathogen identification as *Enterococcus faecium* and antibiotic susceptibility testing, were submitted on day four of treatment. In consequence, screening for a source of infection was extended. Transesophageal echocardiography revealed no indications of endocarditis and urine samples tested as sterile. Magnetic resonance tomography (MRT) of the lumbosacral spine showed multiple fluid accumulations representing small abscesses located inter-, para- and praevertebral between the third lumbar and the first sacral vertebral body (Fig. 1). The patient was transferred to our orthopaedic department and surgical source control with intervertebral abscess removal (L3/L4), posterior interlumbar body fusion (L5/S1) and spondylodesis (L3-S1) was performed on day 12. Antibiotic therapy was broadened by applying intravenous linezolid 600 mg BID.

The patient was dismissed in good general condition without signs of infection on post-operative day 15.

## Microbiological findings

**Fig. 2.**
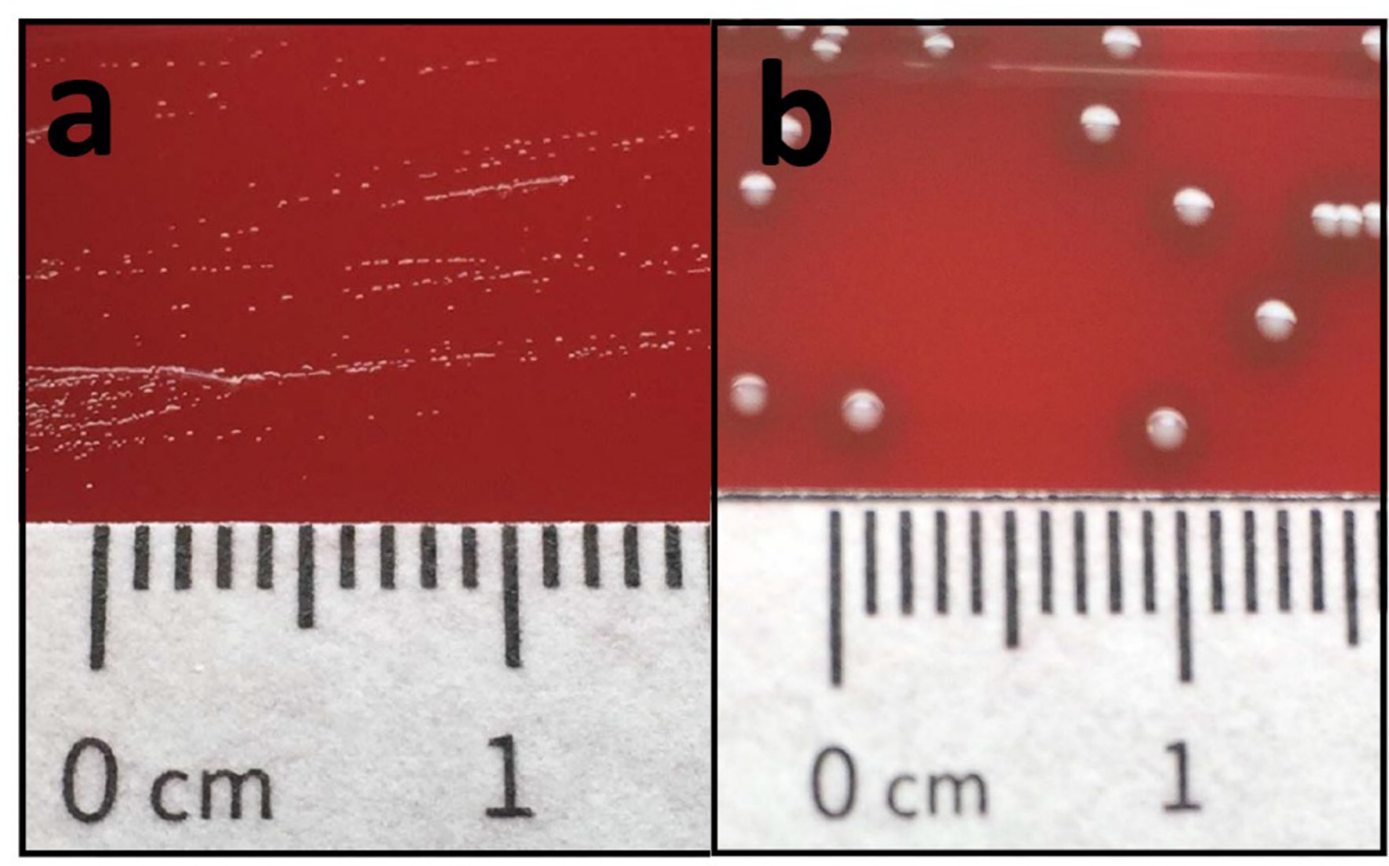
Colony morphology of the isolated *E. faecium* BK001612 SCV strain (a) in comparison to an *E. faecium* wild-type strain (b) (BEI Resources, NIAID, NIH: *Enterococcus faecium *, strain E1162, NR-28979).

Two sets of aerobic/anaerobic blood culture flasks drawn on the day of admission were flagged positive by the automated incubation system (BACTEC 9000; Becton Dickinson, USA) after 30.3 h (minimum) and 35.3 h (maximum) of incubation at 37 °C. All tests performed and results yielded refer to the isolate with the internal identification code BK001612. Microscopic examination of Gram-stained blood culture smears showed Gram-positive cocci in pairs and short chains. Overnight subcultures yielded γ-haemolytic grey colonies that were round and pointy in shape with a diameter of less than 0.1 mm on Columbia agar containing 5 % sheep blood and chocolate agar (both Oxoid, Thermo Fisher Scientific, Germany), as shown in Fig. 2. No growth was observed on Drigalski’s lactose agar (Oxoid, Thermo Fisher Scientific, Germany). The catalase test produced a negative result. Preliminary antibiotic susceptibility testing performed with the BK001612 isolate, applying the agar diffusion method, showed susceptibility to amoxicillin and vancomycin, amongst others.

Due to its colony morphology and antibiotic resistance pattern, the pathogen was misidentified as non-haemolytic streptococci in the first place. However, biochemical identification with the VITEK-2 system (BioMérieux, France) using the VITEK-2 GP ID card resulted in *E. faecium* with excellent test confidence.

The identification results were subsequently confirmed by matrix-assisted laser desorption/ionization time-of-flight (MALDI-TOF) analysis and 16 s rRNA sequencing. For MALDI-TOF analysis, mass spectra were obtained with the VITEK MS system (BioMerieux, France) and compared against the VITEK MS IVD database. 16 s rRNA sequence analysis was performed using the National Center for Biotechnology Information database (NCBI blast). The sequence was deposited in NCBI GenBank under the accession number MK138375.

Automated susceptibility testing performed using the VITEK-2 platform was aborted without results when applying the VITEK-2 AST-ST03 and the AST-P587 test card. A final resistogram was yielded by determining the minimal inhibitory concentrations (MICs) with E-test strips (bestbion dx, Germany) and result categorization according to the European Committee onAntimicrobial Susceptibility Testing (EUCAST) clinical breakpoints version 8.0, as shown in Table 1.

**Table 1.** Antibiotic susceptibility testing results for *E. faecium* SCV BK001612

	Vancomycin	Linezolid	Amoxicillin	Ampicillin/sulbactam	Ciprofloxacin	Gentamicin high-level
MIC	1 mg l^−1^	2 mg l^−1^	0.25 mg l^−1^	0.094 mg l^−1^	6 mg l^−1^	4 mg l^−1^
Interpretation	S	S	S	S	R	S

Single auxotrophism testing was carried out on Mueller–Hinton agar (Oxoid, Thermo Fisher Scientific, Germany) using standard haemin discs and blank discs loaded with 1.5 µg thymidine and 1.5 µg menadione (all Sigma-Aldrich, USA). No switch of phenotype or enhancement of growth was observed around the discs.

## Discussion

We present the first case of multiple spinal abscesses and spondylodiscitis caused by an *E. faecium* SCV.

Of interest, the SCV strain described in our case was susceptible to all of the cell wall active agents tested. This is to some extent exceptional, because in the last three decades high-level ampicillin resistance in clinical *E. faecium* isolates rose to over 90 % in Germany and ampicillin-susceptible strains are only rarely isolated in our laboratory [8]. Further, for bacteria other than enterococci, SCVs are described to be rather more resistant to antibiotics than their related wild-type phenotype. For example, high-level gentamicin resistance is common in *Staphylococcus aureus* SCV strains [9] and increased resistance levels towards beta-lactam antibiotics have also been described for SCVs [10, 11]. The few case reports existing so far report ampicillin-resistant isolates as well as an ampicillin-susceptibile *E. faecium* SCV isolate [3, 7].

Ciprofloxacin resistance as in our case is a common trait in *E. faecium* and is found in the majority of strains isolated in Germany [12]. Subinhibitory dosing of ciprofloxacin has previously been described to induce SCV formation and the expression of adhesion factors facilitating pathogen persistence [13, 14]. Thus, the high ciprofloxacin MIC of the BK001612 *E. faecium* strain might have contributed to expression of the SCV phenotype. However, a prior treatment with ciprofloxacin is not documented in the medical history of this case.

Furthermore, it is relevant to mention the difficulties we encountered in performing automated susceptibility testing (AST) using the VITEK-2 system. The VITEK-2 platform is in widespread use in medical microbiology laboratories and has proven to be reliable for susceptibility testing of enterococci [15]. Nevertheless, in our case AST was aborted repeatedly when using the *E. faecium* SCV BK001612 strain. Most likely this was due to slow growth of SCVs, which was also indicated by the long time-to-positivity of the blood culture flasks, which hinders correct interpretation of growth curves by the VITEK-2 system. Therefore, in cases of suspected SCV infection, manual susceptibility testing using the E-strip test seems to be the preferred approach.

Misidentification of SCVs, as happened in our case, has been described previously. While in previous cases the biochemical profile or single biochemical tests led to wrong conclusions [16–19], in our case the morphology of the bacterial colonies and the antibiotic resistance pattern were misleading characteristics. A similar mistake has been described by Ogihara *et al.,* who misidentified an *E. faecalis* SCV isolate with non-haemolytic streptococci. The authors also emphasized the potential clinical impact of species misidentification through choosing inappropriate antibiotic therapy [5]. Our case gives another good example of SCVs misleading clinical microbiologists and potentially impairing patient care. Therefore, greater awareness among clinical microbiologists of SCVs as a potential phenotype is necessary.

This case provides relevant observations regarding the antibiotic resistance of SCVs and the diagnostic challenges medical microbiologists face when encountering this phenotype. Furthermore, it provides additional proof of the clinical relevance of SCVs in deep-seated infections caused by enterococci.

### Conclusion

In summary, we describe the first case of spondylodiscitis and spinal abscess caused by an *Enterococcus faecium* SCV. The aim of this report is to raise awareness among clinical microbiologists of the small-colony phenotype in enterococci. In particular, when encountering bacterial colonies resembling γ-haemolytic streptococci with contradictory resistance patterns or ambiguous biochemical test results in deep-seated infections, SCVs of enterococci should be taken into consideration.
